# The Silent Pandemic: Antifungal Resistance and the Future of Invasive Fungal Disease Management

**DOI:** 10.3390/microorganisms14030599

**Published:** 2026-03-06

**Authors:** Ruchika Bagga, Kumudhavalli Kavanoor Sridhar

**Affiliations:** 1 Department of Pathobiology and Laboratory Medicine, Schulich School of Medicine and Dentistry, Western University, London, ON N6A 3K7, Canada; 2 Department of Pathobiology and Laboratory Medicine, London Health Sciences Centre, London, ON N6A 5W9, Canada; 3 Department of Laboratory Medicine & Pathobiology, University of Toronto, Toronto, ON M5S 3K3, Canada

**Keywords:** antifungal resistance, *Candidozyma auris*, *Aspergillus fumigatus*, molecular diagnostics, antifungal stewardship

## Abstract

Invasive fungal diseases (IFDs) represent an escalating global health threat, compounded by the rapid emergence of antifungal resistance (AFR). This review synthesizes the contemporary landscape of AFR from clinical and microbiological perspectives, providing actionable insights for clinical practitioners. We examine the epidemiology of critical pathogens, including *Candidozyma auris*, *clonal Candida parapsilosis*, azole-resistant *Aspergillus fumigatus*, and dissect the underlying molecular mechanisms, from genetic mutations in *ERG11* and *cyp51A* to novel emerging epigenetic and adaptive strategies. We critically appraise the diagnostic gap between phenotypic testing and clinical urgency, highlighting the role of rapid molecular assays and next-generation sequencing. Finally, we evaluate evidence-based therapeutic strategies, including the integration of novel agents such as rezafungin, ibrexafungerp, olorofim, and fosmanogepix), while emphasizing the imperative of antifungal stewardship, infection prevention and control in mitigating resistance, and “One-Health” interventions.

## 1. Introduction

The global burden of antimicrobial resistance, long dominated by bacterial pathogens, has expanded to include a formidable mycological crisis [1,2]. The incidence of invasive fungal diseases (IFDs) is rising, driven by a growing population of immunocompromised hosts, advanced medical interventions, and climate-induced ecological shifts [3,4]. Concurrent with this rise is the inexorable development of antifungal resistance (AFR), which threatens to compromise our limited therapeutic armamentarium.

In 2022, the World Health Organization (WHO) published its inaugural Fungal Priority Pathogens List (FPPL) and, in April 2025, released landmark reports highlighting a severe shortage of effective agents and diagnostics [1,2]. Pathogens such as *Candidozyma auris* and azole-resistant *Aspergillus fumigatus* are now categorized as “critical” threats [1]. For the practicing physician, AFR has transitioned from a niche academic concern to a frontline clinical reality, manifesting unexpected treatment failures and complex salvage therapy decisions.

The challenge is multifaceted, spanning delayed diagnostics, a narrow pipeline of novel agents, and the intricate interplay between environmental fungicide use and clinical resistance. This review provides a comprehensive, state-of-the-art summary of the challenges in diagnosing and managing AFR, focusing on the most clinically relevant pathogens and offering evidence-based guidance for navigating this complex landscape.

This narrative review synthesizes evidence on antifungal resistance published between 2019 and 2025. We systematically searched PubMed, Embase, and Web of Science using terms: “antifungal resistance,” “Candida auris,” “Aspergillus fumigatus,” “azole resistance,” “echinocandin resistance,” combined with “epidemiology,” “mechanisms,” “diagnostics,” and “treatment.” We prioritized: (1) national/international surveillance reports from WHO, CDC, and ECDC; (2) multicenter studies with ≥50 isolates; (3) clinical trials evaluating novel antifungal agents; and (4) molecular mechanistic studies. This review emphasizes clinically actionable insights for practicing physicians managing invasive fungal diseases.

## 2. Epidemiology and Surveillance: A Shifting Global Landscape

Effective AFR management begins with robust surveillance. While fungal tracking has historically lagged behind bacterial surveillance, recent global and national initiatives have gained significant momentum [1,2,3,4].

### 2.1. Global and Regional Surveillance Networks

The past five years represent a paradigm shift in coordinated fungal surveillance. In September 2024, the World Health Organization (WHO) formally initiated a data call to integrate antifungal susceptibility data into its Global Antimicrobial Resistance and Use Surveillance System (GLASS), a landmark step toward standardized global reporting [2]. This complements long-standing regional efforts by the US Centers for Disease Control and Prevention (CDC) and the European Centre for Disease Prevention and Control (ECDC) [3,4].

The CDC’s 2024 Antimicrobial Resistance Threats report provides updated national burden estimates, while the ECDC’s 2025 surveillance report now mandates reporting for invasive *Candida* spp., offering unprecedented insight into European longitudinal trends [3,4,5]. Notably, there remains a lack of recent public reporting from large, industry-sponsored networks such as SENTRY and ARTEMIS regarding specific emerging resistance mechanisms. This underscores a growing reliance on national public health agencies and academic multicenter studies to fill critical data gaps [6].

### 2.2. Pathogen-Specific Resistance Trends

#### 2.2.1. *Candidozyma auris*: The Archetypal Multidrug-Resistant Pathogen

*C. auris* remains a preeminent global public health emergency, characterized by rapid nosocomial transmission, environmental persistence, and intrinsic or rapidly acquired multidrug resistance (MDR) [6,7,8,9]. The CDC reported an alarming 95% increase in US clinical cases between 2021 and 2022, with 2377 clinical cases documented in 2024 alone [7]. Epidemiological data from 2025 confirm this trajectory; for instance, Jackson Health System in Miami reported a 590% increase in cases since 2019 [8]. Similarly, the ECDC documented 4012 cases across 15 EU/EEA countries, with regions in Spain and Italy reporting endemicity [4,5,9]. Notably, these prevalence estimates reflect active surveillance in high-resource settings with advanced laboratory capacity. The 95% increase in the U.S. likely represents a combination of true epidemic spread and enhanced detection following the 2018 mandate for national reporting [7].

Resistance profiles are increasingly austere. While fluconazole resistance is nearly universal, the rise in echinocandin resistance is of greater clinical concern. Genomic surveillance in New York City (2020–2024) identified that while isolates remained phylogenetically linked to Clade I, emerging resistance to micafungin and anidulafungin was driven by *FKS1* hotspot mutations (e.g., S639Y and R1354S) [10]. Approximately 12% of US *C. auris* isolates now exhibit echinocandin resistance [2,9].

#### 2.2.2. *Candida parapsilosis*: Clonal Expansion of Azole Resistance

Historically considered susceptible to triazoles, *C. parapsilosis* has undergone a dramatic epidemiological shift toward fluconazole resistance [11]. A seven-year surveillance study in Northern Italy (2018–2024) identified a 73% fluconazole resistance rate among 169 bloodstream isolates [11]. This shift was primarily driven by the Y132F mutation in the *ERG11* gene, with clonal spread confirmed by microsatellite typing. ECDC data from 2025 indicate that fluconazole resistance in *C. parapsilosis* now exceeds 10% in several Southern European countries [5]. Despite reduced biofilm production in these clones, their efficient healthcare-associated transmission often necessitates a therapeutic shift toward echinocandins or polyenes [11].

#### 2.2.3. *Candida albicans*: Relative Stability Amidst Emerging Complexity

In contrast to non-albicans species, *C. albicans* has maintained relatively low rates of clinically significant resistance [6,12]. A 21-year cohort study in Barcelona found that among isolates collected between 2020 and 2025, only 2% were azole-resistant, and none exhibited echinocandin resistance [12]. However, the rare azole-resistant isolates identified harbored complex genetic alterations, including mutations in *ERG11* and its upregulators, signaling a persistent evolutionary capacity that requires continued vigilance [12]. Multicenter data on echinocandin resistance in *C. albicans* remain scarce, representing a key surveillance gap [12].

#### 2.2.4. *Aspergillus fumigatus*: The One Health Environmental-Clinical Nexus

Azole resistance in *A. fumigatus* serves as a primary example of a One Health challenge, linked to the extensive agricultural use of demethylation inhibitor (DMI) fungicides [13]. These environmental pressures select resistance mechanisms that subsequently manifest in clinical settings.

The dominant mechanisms involve tandem repeat (TR) mutations in the *cyp51A* promoter region combined with point mutations [13,14]. A 12-year study in Germany (2011–2022) found that 92% of resistant clinical isolates were resistant to itraconazole and 98% to voriconazole [13]. While the TR34/L98H allele remains dominant (86%), the TR46/Y121F/T289A allele has increased in prevalence since 2016 [13,14,15]. Detection method also influences prevalence: culture-based screening underestimates resistance compared to PCR-based cyp51A screening [16]. Global data locked in 2025 confirm high regional clustering in Germany, South Korea, and China, mirroring local fungicide application patterns [14,15]. This environmental-clinical linkage is further substantiated by a 2024 *Nature* review, which notes that these mutations reduce the efficacy of clinical triazoles like voriconazole and isavuconazole [15]. Consequently, in regions where the prevalence of TR-mediated resistance exceeds 10%, empiric voriconazole monotherapy for invasive aspergillosis is no longer considered a viable primary strategy [13].

Table 1 summarizes the epidemiological shifts, primary molecular drivers, and clinical management adjustments for major fungal pathogens.

### 2.3. The Hidden Burden: Resistance in Low- and Middle-Income Countries

Although much contemporary resistance surveillance originates from North America and Europe, emerging data from Asia, Latin America, and parts of Africa suggest a broader and potentially underrecognized global burden. Reports from Indian intensive care units describe high proportions of *C auris* candidemia (10–30%) [16] characterized by near-universal fluconazole resistance and emerging echinocandin resistance. However, because these data largely derive from tertiary referral centers, they may not represent national prevalence [16].

Similarly, geographically structured, azole-resistant *Aspergillus fumigatus* genotypes have been documented in South Korea and China, supporting the influence of environmental fungicide exposure in resistance selection [15]. Despite these findings, coordinated national surveillance systems remain limited in many low- and middle-income countries (LMICs), where access to routine AFST is inconsistent. As a result, resistance in these regions is likely simultaneously underdetected and underreported, highlighting a substantial global knowledge gap.

## 3. Mechanism of Antifungal Resistance

A comprehensive understanding of resistance mechanisms is essential for interpreting diagnostic results and anticipating therapeutic failure. Resistance in fungal pathogens is a multifaceted phenomenon, categorized as intrinsic (innate insensitivity), acquired (developed via selective pressure), or tolerance (the ability of a subpopulation to grow slowly at drug concentrations exceeding the Minimum Inhibitory Concentration (MIC)) [2,3,4,8].

The molecular landscape of resistance involves altered target affinity, target overabundance, reduced intracellular drug accumulation via efflux transporters, and complex biofilm architectures [4,8]. Recent research into the genetic and epigenetic regulators of these pathways, coupled with cellular stress adaptation, is critical for developing next-generation countermeasures [8].

### 3.1. Core Genetic Mutations

Resistance is primarily driven by stable genomic alterations that interfere with drug-target interactions.

#### 3.1.1. Azole Resistance: Target Alteration and Overexpression

The primary mechanism of azole resistance involves alterations in the ergosterol biosynthesis pathway, specifically targeting the lanosterol 14-α-demethylase enzyme, encoded by *ERG11* in *Candida* and *cyp51A* in *Aspergillus species*.

- Target Site Mutations: Single-nucleotide polymorphisms (SNPs) that reduce drug-binding affinity are common. The Y132F substitution in *C. parapsilosis* is a potent example, conferring high-level fluconazole resistance [11]. In *C. auris*, mutations like Y132F and K143R in *ERG11* are ubiquitous across clades [10].
- Gene Overexpression: Increased enzyme production can titrate out the available drug. This is often achieved through upregulation of transcription factors (e.g., *TAC1b* in *C. auris*) or, in the case of *A. fumigatus*, by inserting tandem repeats (TR34, TR46) in the *cyp51A* promoter region, which dramatically increases gene expression [10,13]
- Efflux Pumps: Overexpression of drug efflux pumps, such as those from the ATP-binding cassette (ABC) superfamily (*CDR1*, *CDR2*) and the major facilitator superfamily (MFS) (*MDR1*), actively expels azoles from the fungal cell. This is a key mechanism in *C. albicans* and contributes to the pan-azole resistance of *C. auris* [10,12]

#### 3.1.2. Echinocandin Resistance: FKS Hotspot Mutations

Echinocandins non-competitively inhibit β-(1,3)-D-glucan synthase, a critical enzyme for cell wall synthesis encoded by the *FKS* genes. Resistance is almost exclusively mediated by acquired point mutations in conserved “hotspot” regions of the *FKS1* gene (or *FKS2* in *C. glabrata*) [10].

- Hotspot Mutations: In *C. auris*, the S639Y and R1354S substitutions in FKS1 are well-characterized causes of resistance, leading to elevated MICs for micafungin and anidulafungin [10]. Emerging mutations such as M690I suggest a diversifying evolutionary landscape of resistance under clinical pressure [10]. These mutations reduce the enzyme’s sensitivity to the drug, requiring higher concentrations for inhibition.

### 3.2. Emerging Epigenetic and Adaptive Mechanisms

Beyond fixed genetic changes, fungi employ dynamic and often reversible strategies to survive antifungal stress [15,17].

#### 3.2.1. Epigenetic Regulation and Virulence

Epigenetics, defined as heritable changes in gene expression without DNA sequence alternation, has emerged as a frontier in AFR. Recent studies in 2024 identified the histone acetyltransferase Gcn5 as a master regulator of multidrug resistance in *C. auris* [18]. Deletion of *GCN5* re-sensitizes resistant isolates to azoles, polyenes, and echinocandins while simultaneously attenuating virulence. Small-molecule inhibition of Gcn5 has demonstrated synergy with caspofungin in murine models, suggesting that targeting the “writers” of the epigenetic code could disable resistance at its transcriptional root [18].

#### 3.2.2. Biofilm-Associated Tolerance

Biofilms exhibit a form of adaptive tolerance that allows fungal communities to withstand drug concentrations up to 100-fold higher than their planktonic counterparts [15]. This biofilm-mediated tolerance is a major cause of treatment failure in device-associated infections and highlights the need for therapies that can disrupt the biofilm structure or target persister cells [8,11]. This is a multifactorial emergent property rather than a result of stable mutations:

- Matrix Sequestration: The extracellular matrix (ECM), particularly β-glucans, physically sequesters antifungal drugs, preventing penetrance to the deeper cell layers [8,11].
- Efflux Pump Upregulation: Cells within the biofilm environment upregulate efflux pumps, contributing to a high-level resistance phenotype [8].
- Metabolic Quiescence: A subpopulation of “Persister” cells enters a dormant metabolic state, rendering them immune to drugs that target active cellular processes [11].
- Stress Response Activation: The biofilm environment triggers high-level activation of heat-shock proteins (Hsp90) and calcineurin pathways, which coordinate cellular repair and prevent apoptosis, enhancing survival under drug-induced stress [8,11,15].

## 4. Diagnostic Challenges: The Race Against Time

The efficacy of any therapeutic strategy is contingent upon accurate and timely diagnosis. In clinical mycology, the diagnostic workflow is fraught with challenges that create a critical disconnect between the onset of infection and the initiation of targeted therapy.

### 4.1. The Pre-Analytical Barrier: Sample Acquisition

The diagnostic race is often lost before the sample even reaches the laboratory. Obtaining high-quality, non-contaminated specimens from the site of infection remains a formidable obstacle:

- Invasiveness versus Yield: IFDs frequently involve deep-seated tissues (e.g., lung parenchyma, brain, or intra-abdominal space). Obtaining a definitive sample often requires invasive procedures such as ultrasound-guided fine-needle aspiration (FNA), bronchoalveolar lavage (BAL), or open-tissue biopsy. In critically ill or thrombocytopenic patients, these procedures may be contraindicated due to the high risk of hemorrhage or clinical instability [6,12].
- Contamination and Commensalism: Differentiating between colonization and true infection is particularly difficult with *Candida* and *Aspergillus* species, which can be part of the normal flora or common environmental contaminants. Non-sterile sample collection (e.g., sputum or superficial swabs) often yields misleading results, leading to either over-treatment or misidentification of the true causative pathogen [5,13].
- Low Fungal Burden: Even in disseminated infections, the concentration of fungal cells in peripheral blood is often extremely low (often <10 CFU/mL), contributing to the notoriously low sensitivity (approx 50%) of standard blood cultures [12]. This “needle in a haystack” scenario means that even when a sample is obtained, the diagnostic yield may be insufficient for subsequent AFST [16,19].

### 4.2. Barriers to Timely and Accurate Diagnosis

The identification of antifungal resistance (AFR) remains a primary hurdle in clinical practice due to several systemic factors:

- Lag in Culture-Based Methods: The current “gold standard” relies on phenotypic Antifungal Susceptibility Testing (AFST) via CLSI or EUCAST methodologies [5,6]. These growth-dependent assays are inherently slow, often requiring several days for yeasts and over a week for filamentous fungi, delaying targeted treatment in critically ill patients.
- Infrastructure and Expertise Gaps: Performing AFST for molds requires specialized mycological expertise and equipment often absent in community hospitals. The necessity of referring samples to centralized reference laboratories further prolongs turnaround times (TAT) [5,6]
- The “Breakpoint” Vacuum: For many emerging pathogens and novel antifungal agents, standardized clinical breakpoints (CBPs)—which correlate in vitro MICs with clinical outcomes—have not been established. In these instances, laboratories must rely on Epidemiological Cutoff Values (ECVs), which identify non-wild-type isolates but do not necessarily predict clinical failure. Currently, CLSI breakpoints for molds are limited, notably only existing for voriconazole against *Aspergillus fumigatus sensu stricto* [5,6].
- Limitations of Culture-Independent Diagnostics (CIDs): While assays for biomarkers (e.g., beta-D-glucan, galactomannan, and lateral flow assays) facilitate earlier detection of fungal presence, they lack the specificity required to provide data on drug resistance profiles [6]
- No standard molecular diagnosis guidance: Molecular methods (e.g., PCR for resistance genes like cyp51A mutations in *A. fumigatus*) exist but are not yet standardized or widely implemented. Mostly used for research purposes or available in reference laboratories with limitations.

### 4.3. The Turnaround-Time vs. Clinical Decision-Making Dilemma

The central challenge for clinicians remains the conflict between turnaround time (TAT) and the urgency of clinical intervention. An invasive fungal disease (IFD) in a critically ill patient constitutes a medical emergency; however, the 5- to 7-day TAT required for mold susceptibility results is clinically untenable [13,16]. This delay necessitates prolonged reliance on broad-spectrum empiric regimens, which may be inappropriate in the face of emerging multidrug-resistant (MDR) strains [1,2,3,4,5].

While molecular techniques such as PCR offer a substantial kinetic advantage, their targeted nature is a significant limitation. These assays can only detect specific, “canonical” mutations. Consequently, a negative PCR result for *FKS* mutations does not definitively exclude resistance, as it may be driven by:

- Non-canonical mutations not covered by the primer set [10,14].
- Epigenetic modifications (e.g., *GCN5*-mediated regulation) [20].
- Adaptive responses such as mitochondrial stress signaling or cell wall remodeling [8,15].

### 4.4. Critical Appraisal of AFST Platforms

The central dilemma for clinicians is the trade-off between turnaround time and diagnostic depth. Table 2 summarizes the current and emerging methodologies for detecting antifungal resistance.

#### Challenges in AFST Interpretation

Interpretation of antifungal susceptibility results remains complex and, in certain contexts, controversial. Clinical breakpoints differ between CLSI and EUCAST frameworks, and for many mold–drug combinations, validated clinical breakpoints are lacking, necessitating reliance on epidemiologic cutoff values (ECVs) [23]. While ECVs are useful for identifying non-wild-type isolates, they do not reliably predict clinical outcome.

Furthermore, outcome-correlated MIC data for invasive mold infections remain limited, complicating interpretation in individual patients. These limitations underscore the importance of integrating AFST results with host factors, pharmacokinetic/pharmacodynamic (PK/PD) considerations, and local epidemiology rather than relying solely on categorical susceptibility designations.

Some Candida isolates exhibit increased growth at high echinocandin concentrations in vitro. While dismissed by some as artifact, animal models demonstrate reduced efficacy at supratherapeutic doses [24]. Clinicians remain uncertain whether dose escalation benefits or harms patients with paradoxical isolates.

### 4.5. Bridging the Gap: Recent Progress

Recent advances aim to mitigate these delays. Commercial broth microdilution (BMD) panels utilizing digital imaging can now provide 24 h MICs for common yeasts, significantly improving reproducibility [23]. Furthermore, the expansion of the CDC-FDA Antibiotic Resistance Isolate Bank provides a crucial resource for well-characterized isolates for validating new molecular assays [25].

Despite these gains, the “ideal” diagnostic—one that is rapid, comprehensive, and point-of-care, remains elusive. For instance, while molecular methods like PCR offer speed, their targeted nature may miss novel or non-canonical resistance mechanisms, such as epigenetic modifications or metabolic tolerance.

## 5. Implementation of AFST: A Stepwise Approach for Resource-Constrained Settings

Sustainable implementation of Antifungal Susceptibility Testing (AFST) in Resource-Constrained Settings (RCS) requires a strategy that balances technical capability with infrastructure and clinical utility [2,19]. The following seven-step approach prioritizes feasibility and high-impact interventions.

Step 1: Accurate Species Identification (The Crucial First Focus)

The most resource-efficient intervention is ensuring reliable species-level identification for a prioritized list of pathogens.

- Rationale: Identification allows clinicians to predict intrinsic resistance (Table 3), facilitating immediate therapeutic adjustments even before AFST results are available. This is particularly vital for “cryptic species” within the *Aspergillus* complexes (e.g., *A. lentulus*, *A. calidoustus*, or *A. tubingensis*), which often exhibit high MICs to triazoles [13,14].
- Techniques: Laboratories should utilize MALDI-TOF MS where available, as current libraries (e.g., Bruker, VITEK with supplementary database like MSI-2 or in house libraries) are increasingly robust for both yeasts and filamentous fungi [17,18].

Step 2: Clear Protocols and Standardization

Development and adoption of internationally recognized protocols are essential.

- Action: Implement Standard Operating Procedures (SOPs) for specimen collection, and specialized fungal media (e.g., supplemented with antibacterials). Standardize the methodology (e.g., disk diffusion or gradient strips) to ensure reproducibility and comparability with global surveillance data [23].
- Goal: To ensure consistent and reproducible results, minimizing variation and improving comparability with global data. Many commercially available testing (frozen or dry yeast) panels are available.

Step 3: Prioritization of Pathogens and Available Agents

AFST should be limited to medically important fungi isolated from sterile sites and only to the antifungal agents available within the local formulary [6].

- Action: Limit AFST to medically important fungi (yeasts and *Aspergillus* spp. from probable/proven invasive diseases) and only to the antifungal agents available in the country and which have clinical breakpoints from regulatory body (Table 4). Testing drugs that are unavailable locally is of little clinical value.
- Goal: To maximize the clinical relevance of limited testing capacity.

Step 4: Access to Quality Media and Reagents

Establishing a consistent supply chain is a fundamental operational challenge.

- Action: Decisions on technology (e.g., manual broth microdilution vs. commercial panels) must balance cost with the need for high-quality, standardized media that minimize MIC variability [17].
- Goal: To ensure the stability of the diagnostic pipeline. Inconsistent media quality is a leading cause of “MIC drift,” which can result in the misclassification of isolates and inappropriate clinical decisions.

Step 5: Maintenance of Quality Control (QC) Strains

Accuracy must be verified daily to maintain clinical trust.

- Action: Acquire reference strains (e.g., ATCC) to verify assay performance. This ensures both quantitative precision (MIC ranges) and qualitative accuracy (Sensitive/Intermediate/Resistant categorization) [23,25].
- Goal: To ensure the reliability of the AFST service, a critical prerequisite for clinical trust. Reference strains provide the “standard meter” against which clinical isolates are measured, ensuring that a “Resistant” result in a regional lab matches the definition in a reference center.

Step 6: Laboratory and Clinical Training

The “bench-to-bedside” link is vital.

- Action: Train laboratory personnel in morphological and molecular AFST techniques, and train clinical staff in Antifungal Stewardship (ASP)—specifically on how to use AFST data for therapeutic de-escalation [26,27].
- Goal: To build the local expertise necessary for sustainability and to strengthen the link between the lab and the clinical team.

Step 7: External Quality Assurance (EQA) and Surveillance

Reliability must be periodically validated externally, and the data must be utilized systemically [2,5,11,12].

- Action 7a (EQA): Participate in external proficiency testing to validate laboratory competence.
- Action 7b (Surveillance): Aggregate AFST data to establish local resistance maps.
- Goal: This data is essential for informing local empirical guidelines and strengthening ASPs. By identifying local trends (e.g., a rise in *C. parapsilosis* azole resistance), the laboratory transforms from a service provider into a strategic partner in infection control.

## 6. Therapeutic Strategies: Navigating a Shifting Armamentarium

The management of resistant fungal infections requires a nuanced approach, balancing the utility of established drug classes with the judicious integration of novel therapies.

### 6.1. Evidence-Based Use of Existing Antifungals

- Azoles: Despite rising resistance, triazoles remain fundamental. Fluconazole is the preferred agent for susceptible *Candida* infections [6]. Voriconazole and isavuconazole remain the cornerstones for invasive aspergillosis (IA), though their empiric use must be informed by local *A. fumigatus* resistance maps [13,14]. Posaconazole, available in both IV and gastro-resistant tablet formulations, remains a primary choice for prophylaxis and step-down therapy [1,2].
- Echinocandins: This class is the first-line treatment for most invasive candidiasis (IC) [25]. However, the emergence of *FKS*-mutant *C. auris* poses a significant threat to this class [10]. For *Aspergillus*, echinocandins are fungistatic and typically reserved for salvage or combination therapy [20,26].
- Polyenes: Liposomal amphotericin B (L-AmB) retains the broadest spectrum of activity with no widespread, mechanism-defined resistance. It remains the “gold standard” for salvage therapy, severe IFDs of unknown etiology, and multidrug-resistant (MDR) pathogens, including echinocandin-resistant *C. auris* or azole-resistant molds [1,5,28].

### 6.2. The Emerging Antifungal Pipeline

The period from 2019 to 2025 has seen a significant expansion of the antifungal pipeline, introducing several first-in-class agents designed to bypass current resistance mechanisms (Table 5 summarizes the clinical trial data and therapeutic niches for the newest generation of antifungal agents).

The arrival of these agents represents a paradigm shift in the management of invasive fungal disease (IFD). The long half-life of rezafungin simplifies the logistical burden of candidemia treatment, potentially reducing healthcare costs by facilitating Outpatient Parenteral Antifungal Therapy (OPAT) and earlier hospital discharge [29,30,32,33,34,35].

Furthermore, ibrexafungerp provides the first oral option for *Candida* species harboring *FKS* mutations, filling a critical void left by echinocandin resistance [30,31]. For the first time, patients with highly resistant mold infections—such as those caused by *Lomentospora* or triazole-resistant *Aspergillus*—have targeted options in olorofim and fosmanogepix [28,32]. These agents offer a lifeline for cases previously considered untreatable, though their integration will require vigilant monitoring for emerging resistance and secondary side effects [28,33]. Immunosuppression severity significantly modifies resistance-associated mortality. Patients with profound neutropenia (<100 cells/μL) show minimal survival benefit from echinocandins versus azoles for susceptible *Candida*, as host defenses cannot synergize with fungistatic activity [26,36]. Other pharmacodynamic considerations include azole CNS penetration (fluconazole achieves 70–90% CSF levels) and tissue-specific drug concentrations (lipid-associated amphotericin achieves high lung but poor urinary levels)

### 6.3. Salvage, Combination, and Step-Down Therapies

For patients failing initial monotherapy, combination therapy is an increasingly viable strategy. A meta-analysis of salvage therapy for IA suggested that combining an echinocandin with either an azole or L-AmB improved 12-week survival compared to monotherapy [20]. The advent of novel agents is prompting a re-evaluation of these strategies, with trials currently exploring Ibrexafungerp plus Voriconazole for IA [26].

However, the role of combination antifungal therapy remains debated. Although meta-analytic data suggest potential survival benefit in salvage therapy for invasive aspergillosis when an echinocandin is combined with either a triazole or liposomal amphotericin B, these analyses are largely based on observational or heterogeneous studies [20]. Contemporary reviews emphasize the absence of definitive randomized controlled trial evidence supporting routine upfront combination therapy and highlight concerns regarding toxicity, cost, and drug–drug interactions [26]. As such, combination therapy should generally be reserved for refractory disease, high fungal burden, or documented resistance, pending stronger prospective data.

The transition from IV to oral therapy aka “step-down therapy” is a cornerstone of ASP. The oral availability of Ibrexafungerp and Fosmanogepix, alongside established triazoles, will be critical for optimizing treatment duration, reducing hospital stays, and facilitating OPAT [26,27,29].

## 7. Stewardship and Infection Control: The Twin Pillars of Resistance Mitigation

Therapeutic innovation alone is insufficient to combat AFR. The long-term efficacy of the antifungal armamentarium depends on the synergy between Antifungal Stewardship (AFS) and Infection Prevention and Control (IPC) [3,27]. This synergy forms the core of a modern “One Health” approach [15].

### 7.1. Antifungal Stewardship (AFS)

The principles of antimicrobial stewardship are increasingly tailored to the unique challenges of fungal disease. The CDC’s “Core Elements” framework now guides AFS implementation, emphasizing the tracking of antifungal-specific metrics alongside traditional antibacterials [3,27].

- Data-Driven Intervention: Modern AFS programs leverage rapid diagnostics to move beyond empiric therapy. The integration of MALDI-TOF MS [21,22] and PCR for resistance markers (e.g., *cyp51A* or *FKS*) [10,14] allows stewardship teams to recommend targeted de-escalation or escalation within 24 h of culture positivity.
- Barriers to Implementation: Key obstacles include a global shortage of mycology-specific expertise on stewardship teams and the lack of standardized, risk-adjusted metrics for antifungal consumption (e.g., Days of Therapy (DOT) vs. Defined Daily Doses (DDD)).

### 7.2. Infection Prevention and Control (IPC)

IPC is the primary defense against the nosocomial transmission of resistant fungi, particularly *C. auris*. Outbreak investigations have demonstrated that standard contact precautions are often insufficient to halt transmission in high-acuity settings [9,27].

The CDC-Recommended Multi-pronged Strategy [27]:

1. Aggressive Screening: Real-time PCR screening of high-risk patients to enable early cohorting and isolation [10].
2. Contact Precautions: Strict adherence to gown and glove protocols for all patient encounters.
3. Environmental Decontamination: Use of sporicidal disinfectants (e.g., List P-registered products); standard quaternary ammonium compounds are often ineffective against *C. auris*.
4. Inter-facility Communication: Standardized notification protocols during patient transfer to prevent regional endemicity.

## 8. Future Directions: Innovations on the Horizon

The fight against AFR is entering a high-tech phase, characterized by point-of-care diagnostics and immunoprophylaxis.

### 8.1. Next-Generation Therapeutics and Vaccines

- Inhaled Strategies: Opelconazole, an inhaled triazole, has shown promise in Phase II trials for preventing pulmonary aspergillosis in lung transplant recipients. This lung-targeted approach minimizes systemic toxicity and drug–drug interactions [37]
- mRNA Vaccines: Leveraging mRNA-lipid nanoparticle (LNP) technology, a 2024 proof-of-concept study demonstrated 100% protection against lethal *Cryptococcus* in murine models [38]. This represents a potential breakthrough in preventing infections in immunocompromised populations.

### 8.2. AI and Rapid Diagnostics

- CRISPR-based Diagnostics: Platforms such as the RID-MyC assay can detect fungal DNA in under one hour, meeting the WHO’s “ASSURED” criteria for resource-limited settings [39].
- Artificial Intelligence (AI): Deep learning models are now achieving >90% accuracy in diagnosing fungal keratitis from microscopy images. The use of “Explainable AI” (XAI) helps build clinician trust by highlighting the specific morphological features driving the diagnosis [40].
- Clinical Implications: A Decision-Making Framework

Antifungal resistance has transitioned from a theoretical concern to a clinically actionable threat that directly influences empiric and targeted management of invasive fungal diseases [41,42]. Clinicians must integrate local epidemiologic data, host risk factors, and pharmacokinetic/pharmacodynamic principles when initiating therapy. In regions where azole-resistant *Aspergillus fumigatus* prevalence exceeds 10%, empiric voriconazole monotherapy may be inadequate, and alternative or combination strategies should be considered [13,14,15]. Similarly, increasing echinocandin resistance in *C auris* necessitates early species identification and prompt susceptibility testing to guide therapy escalation [10].

Interpretation of antifungal susceptibility testing should not rely solely on categorical “susceptible” or “resistant” designations. Differences between CLSI and EUCAST breakpoints, absence of mold breakpoints for several agents, and reliance on epidemiologic cutoff values require clinical correlation [23]. Molecular assays may accelerate detection of common resistance mechanisms but cannot exclude non-canonical mutations, epigenetic regulation, or tolerance phenotypes; phenotypic confirmation remains essential [10,14,18].

Therapeutic drug monitoring for triazoles, attention to drug–drug interactions, and careful evaluation of immunologic status are critical in preventing selection of resistant subpopulations [13,16,17]. Combination therapy should generally be reserved for salvage settings or documented resistance, pending stronger prospective evidence [20,26].

Ultimately, optimal management requires close collaboration between clinicians, microbiology laboratories, and antifungal stewardship programs. Timely diagnostics, contextual interpretation of susceptibility data, and judicious integration of emerging antifungal agents are essential to preserving therapeutic efficacy and improving patient outcomes in the era of expanding antifungal resistance.

## 9. Conclusions

Antifungal resistance is a clear and present danger to global health, complicating the management of life-threatening infections. The global spread of MDR pathogens like *C. auris* and azole-resistant *A. fumigatus* demands a coordinated response from clinicians, microbiologists, and public health authorities. While the challenges—from diagnostic delays to a historically sparse pipeline—are significant, the landscape is shifting.

The advent of novel antifungal classes, the integration of molecular diagnostics into stewardship, and the potential of mRNA vaccines and AI-driven tools offer a new trajectory. For the clinician, success depends on a commitment to staying abreast of local epidemiology, understanding the nuances of new diagnostic and therapeutic options, and championing the principles of stewardship and infection control. By integrating these advancing frontiers into practice, we can improve patient outcomes and preserve the efficacy of our antifungal armamentarium for the future.

## Figures and Tables

**Table 1:** Summary of Recent (2019–2025) Antifungal Resistance Trends in Key Pathogens.

Pathogen	Key Resistance Phenotype	Prevalence/Trajectory	Dominant Molecular Mechanisms	Clinical Implication
*Candidozyma auris*	Pan-azole resistance; Emerging echinocandin resistance	>95% rise in US cases (2021–22); Endemic in parts of EU. ~12% of US isolates are echinocandin-R [4]	*ERG11* & *TAC1b* mutations (azole-R); *FKS1* hotspot mutations (S639Y, R1354S) (echinocandin-R) [10]	Empiric fluconazole is contraindicated. Echinocandin susceptibility must be confirmed; consider alternative agents for refractory infections.
*Candida parapsilosis*	High-level fluconazole resistance	>70% in regional outbreaks (e.g., Northern Italy); >10% in Southern Europe [11,14]	Clonal spread of strains with *ERG11* Y132F mutation [11]	Fluconazole is unreliable for empiric therapy in high-prevalence regions. Echinocandins are the preferred first-line agents.
*Candida albicans*	Low-level azole and echinocandin resistance	Azole-R ~2%; Echinocandin-R remains rare in bloodstream isolates [11]	Point mutations in *ERG11* and upregulation of efflux pumps (*CDR1*) [12]	Fluconazole and echinocandins remain highly active for most infections, but susceptibility testing is prudent in cases of prior exposure or treatment failure.
*Aspergillus fumigatus*	Pan-azole resistance	>90% resistance to itraconazole/voriconazole in resistant isolates in parts of Europe [13]	Environmentally derived *cyp51A* mutations: TR34/L98H and TR46/Y121F/T289A [14]	Empiric voriconazole monotherapy is inadequate for IA in high-prevalence areas (>10%). Consider combination therapy or novel agents.

Abbreviations: IA, invasive aspergillosis; MDR, multidrug-resistant; R, resistant; TR, tandem repeat; EU, European Union; US, United States. Note: Resistance trajectories are based on data consolidated from CDC (2024) [3], ECDC (2025) [5], and WHO GLASS reports. Molecular mechanisms highlighted (e.g., *FKS1*, *ERG11*, *cyp51A*) represent the most frequently identified markers in recent genomic surveillance studies.

**Table 2:** Critical Appraisal of Antifungal Susceptibility Testing (AFST) Platforms.

Platform	Principal Challenges	Typical TAT	Clinical Impact
**Phenotypic BMD (CLSI/EUCAST)**	Slow growth; Subjective endpoint reading (e.g., “trailing” in azoles, “paradoxical effect” in echinocandins) [17]	2–4 days (yeasts); ≥5–7 days (molds)	Long TAT necessitates prolonged empiric therapy, increasing toxicity risk, cost, and selection pressure.
**MALDI-TOF MS-based AFST**	Requires standardized algorithms and extensive spectral libraries; currently remains largely in research phase [21]	3–6 h (yeasts); 6–24 h (molds)	High potential for rapid resistance detection (e.g., echinocandin-R in *C. auris*); awaiting clinical standardization [22]
**PCR-based Resistance Assays**	Target-limited; can only detect known mutations (e.g., *FKS*, *cyp51A*). Negative result do not rule out alternate mechanisms	2–8 h	Excellent for “ruling in” resistance to guide rapid deescalation; vital for screening and outbreak investigation (*C. auris*).
**Next-Generation Sequencing (NGS)**	High cost; complex bioinformatic and pipeline requirements; necessitates validated mutation databases [19]	1–2 days	Offers the most comprehensive ‘resistome’ view; essential for detecting both known and novel mutations and mixed populations. Increasingly used for outbreak analysis and refractory cases [14]

**Abbreviations: AFST**, antifungal susceptibility testing; **BMD**, broth microdilution; **CLSI**, Clinical and Laboratory Standards Institute; **EUCAST**, European Committee on Antimicrobial Susceptibility Testing; **MALDI-TOF MS**, matrix-assisted laser desorption/ionization–time of flight mass spectrometry; **MIC**, minimum inhibitory concentration; **NGS**, next-generation sequencing; **TAT**, turnaround time. **Note:** Typical TAT represents the time from isolate availability (for phenotypic methods) or specimen acquisition (for molecular methods) to the reporting of results. Clinical impact assessment is based on the ability of the platform to influence real-time therapeutic adjustments in invasive fungal disease (IFD) management.

**Table 3:** Intrinsic Antifungal Resistance in Key Fungi.

Antifungals	Fungal Species	Clinical Context
Fluconazole	*Candida krusei (P. kudriavzevii)*, *C. auris*, *Aspergillus* spp., *Rhodotorula* spp., *Lomentospora prolificans*	Empiric fluconazole is ineffective; *Mucorales* are also resistant to most azoles except posaconazole/isavuconazole.
Echinocandins	*Cryptococcus* spp., *Rhodotorula* spp., *Trichosporon* spp., *Mucorales*	Lack the target enzyme beta (1,3)-D-glucan synthase or possess resistant cell wall structures.
Amphotericin B	*Aspergillus terreus*, *L. prolificans*, *Purpureocillium lilacinum*	High-level resistance: polyenes should be avoided.
Voriconazole	*Mucorales*, *Rasamsonia* spp., Cryptic *Aspergillus* spp.	Often leads to treatment failure in invasive mold infections.

**Table 4:** Availability of Clinical Breakpoints (CBPs) by Regulatory Body.

Fungal Species	CLSI Breakpoints	EUCAST Breakpoints
*Candida albicans*	Fluconazole, Voriconazole, Echinocandins	Fluconazole, Voriconazole, Echinocandins, 5- Flucytosine, Amphotericin B
*Candida glabrata* *	Fluconazole (SDD *), Echinocandins	
*Candida parapsilosis* (sensu stricto)		
*Candida tropicalis*		
*Candida krusei* (*Pichia kudriavzevii*)	Intrinsic Resistance (IR) to Fluconazole (No CBP), Echinocandins	IR to Fluconazole (No CBP), Echinocandins, Amphotericin B
*Candida auris*	Echinocandins, Fluconazole, Amphotericin B (CLSI supplement M60)	Echinocandins, Flucytosine, Amphotericin B (EUCAST documents)
*Cryptococcus neoformans*	Fluconazole, Flucytosine (Limited to Flu/5FC)	Fluconazole, Flucytosine, Amphotericin B
*Aspergillus fumigatus* (sensu stricto)	Voriconazole, Itraconazole, Posaconazole	Voriconazole, Itraconazole, Posaconazole, Isavuconazole, Amphotericin B, Echinocandins
*Aspergillus flavus*	Voriconazole, Itraconazole, Posaconazole (Limited)	Voriconazole, Itraconazole, Posaconazole, Isavuconazole
*Aspergillus terreus*	Voriconazole (Limited)	
*Aspergillus niger*		

* SDD: Susceptible-Dose Dependent. Clinical and Laboratory Standards Institute (CLSI) and the European Committee on Antimicrobial Susceptibility Testing (EUCAST).

**Table 5:** Key Novel Antifungal Agents with Recent Clinical Evidence.

Agent (Class/MoA)	Key Clinical Evidence	Indication/Population	Efficacy & Safety Signals
Rezafungin (Next-gen Echinocandin)	ReSTORE Phase 3: Non-inferior to caspofungin for Day 30 mortality (25.2% vs. 24.8%) [29].	Candidemia and Invasive Candidiasis (IC)	Once-weekly IV dosing; facilitates outpatient therapy. Safety profile comparable to other echinocandins. FDA approved in 2023.
Ibrexafungerp (Triterpenoid; Glucan Synthase Inhibitor)	CANDLE/SCYNERGIA Phase 3: Superior cure rates in VVC; shows activity in refractory IC [30,31].	Recurrent VVC; Candidemia & IC (including *C. auris*)	First oral glucan synthase inhibitor. Retains activity against most echinocandin-resistant *Candida* due to distinct binding sites. Generally well-tolerated with mild GI side effects
Olorofim (Orotomide; DHODH Inhibitor)	Phase 2b (Olorofim-001): 28.7% global success in refractory cases; 11.9% all-cause mortality [28].	Refractory IA & Rare Molds (e.g., *Lomentospora*)	First-in-class mechanism targeting pyrimidine synthesis. Effective against azole-resistant *Aspergillus*. Manageable hepatotoxicity (transaminase elevation in ~10%).
Fosmanogepix (Gwt1 Inhibitor)	AEGIS Phase 2: 40% global success in limited-option invasive mold disease [32].	Invasive Mold Disease; Candidemia	Pro-drug with high bioavailability. Targets fungal protein anchoring. Broad spectrum with minimal CYP3A4 interactions.

**Abbreviations: MoA**, mechanism of action; **IC**, invasive candidiasis; **IA**, invasive aspergillosis; **VVC**, vulvovaginal candidiasis; **RCT**, randomized controlled trial; **DHODH**, dihydroorotate dehydrogenase; **Gwt1**, glycosylphosphatidylinositol-anchored wall protein 1. **Notes:** Efficacy data are derived from primary endpoint analyses of the ReSTORE (Rezafungin), CANDLE (Ibrexafungerp), Olorofim-001 (Olorofim), and AEGIS (Fosmanogepix) trials. Safety signals represent the most frequent treatment-emergent adverse events (TEAEs) reported in Phase 2/3 cohorts.

## Data Availability

No new data were created or analyzed in this study. Data sharing is not applicable.
